# Necrotizing enterocolitis: controversies and challenges

**DOI:** 10.12688/f1000research.6888.1

**Published:** 2015-11-30

**Authors:** Augusto Zani, Agostino Pierro

**Affiliations:** 1 Division of General and Thoracic Surgery, University of Toronto, The Hospital for Sick Children, Toronto, Canada

**Keywords:** Gut, necrotizing enterocolitis, bowel

## Abstract

Necrotizing enterocolitis is a devastating intestinal disease that affects ~5% of preterm neonates. Despite advancements in neonatal care, mortality remains high (30–50%) and controversy still persists with regards to the most appropriate management of neonates with necrotizing enterocolitis. Herein, we review some controversial aspects regarding the epidemiology, imaging, medical and surgical management of necrotizing enterocolitis and we describe new emerging strategies for prevention and treatment.

## Introduction

**Figure 1.**
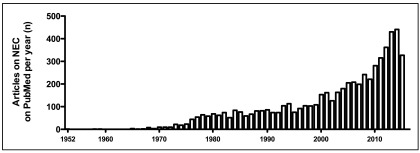
Number of articles published per year on PubMed since 1952 to 2015 (July).

Necrotizing enterocolitis is an inflammatory intestinal disorder primarily seen in premature infants, characterized by variable damage to the intestinal tract, ranging from mucosal injury to full-thickness necrosis and perforation. Reports of infants suffering from necrotizing enterocolitis are found in literature dating back to the nineteenth century ^1^, but its pathology was described only in 1952, when Schmidt and Quaiser labeled it as *enterocolitis ulcerosa necroticans* ^2, 3^. Necrotizing enterocolitis became widely known in the 1960s following an epidemic occurring at the Babies Hospital in New York City between 1955 and 1966 ^4^. The number of neonates collectively treated in New York helped to define the radiological and clinical presentation of this disease. Since then, the interest in and research into necrotizing enterocolitis has risen over the years ( Figure 1), and several randomized controlled trials have been conducted to question or define various aspects of prevention and treatment of this condition. However, controversy still persists with regards to the most appropriate management of neonates with necrotizing enterocolitis. Herein, we review some controversial aspects regarding the epidemiology, imaging, medical and surgical management of necrotizing enterocolitis and we describe new emerging strategies for prevention and treatment.

## Epidemiology

Necrotizing enterocolitis is considered to be the most common gastrointestinal emergency among neonates and affects primarily preterm infants. However, the true incidence of necrotizing enterocolitis is unknown, as it is difficult to establish and identify “mild” or “initial” cases, which correspond to stage I Bell’s classification [ Table 1] ^5, 6^. For this reason, solid epidemiology data are lacking.

**Table 1.** Bell staging criteria for necrotizing enterocolitis, modified from Walsh and Kliegman ^6, 47.^

Stage	I	IIA	IIB	IIIA	IIIB
Description	Suspected NEC	Mild NEC	Moderate NEC	Severe NEC	Severe NEC
Systemic signs	Temperature instability, apnea, bradycardia	Similar to stage I	Mild acidosis, thrombocytopenia	Respiratory and metabolic acidosis, mechanical ventilation, hypotension, oliguria, DIC	Further deterioration and shock
Intestinal signs	Increased gastric residuals, mild abdominal distension, occult blood in the stool	Marked abdominal distension ± tenderness, absent bowel sounds, grossly bloody stools	Abdominal wall edema and tenderness ± palpable mass	Worsening wall edema with erythema and induration	Evidence of perforation
Radiographic signs	Normal or mild ileus	Ileus, dilated bowel loops, focal pneumatosis	Extensive pneumatosis, early ascites ± PVG	Prominent ascites, fixed bowel loop, no free air	Pneumoperitoneum

DIC, disseminated intravascular coagulopathy; NEC, necrotizing enterocolitis; PVG, portal venous gas.

In 2010, Rees *et al.* reported the results of a national study based on a prospective cross-sectional survey administered to 158 level 2 and 3 neonatal intensive care units in the UK between 2005 and 2006 ^7^. A total of 211 infants were diagnosed with necrotizing enterocolitis (45% Bell’s stage I, 21% stage II, and 33% stage III) for a period prevalence of 2% intensive care unit admissions. Data from the Canadian Neonatal Network reported that necrotizing enterocolitis (stage II and III) has an incidence of 5.1% in Canadian infants with a gestational age <33 weeks ^8^. The incidence of necrotizing enterocolitis has increased in recent decades in Canada and the UK due to more preterm and low birth weight infants being born—a trend that will continue as more preterm infants are treated in neonatal intensive care units.

The development of necrotizing enterocolitis varies also according to geographical and ethnic distribution, with lower frequencies in Japan, Switzerland, and Austria, and higher frequencies in Northern America, the UK and Ireland ^8– 14^. It is still unknown whether this variability is due to actual genetic and/or environmental factors in the population or whether it is influenced by neonatal care strategies and/or ethnic background.

The overall mortality rate for necrotizing enterocolitis remains high (30–50%), despite advancements in neonatal care. Moreover, a significant proportion of the survivors, particularly those with stage III necrotizing enterocolitis, have profound neurodevelopmental delay ^14^, resulting in reduced quality of life for the patient and family and in significant costs of ongoing treatment, calculated at between $500 million and $1 billion per year in the US ^15^. The serious and life-long challenges necrotizing enterocolitis places on patient, family, and society indicate that therapeutic strategies to preserve and/or reconstitute the intestinal structure of necrotizing enterocolitis-affected neonates are urgently needed.

## Etiology

Although the exact etiology of necrotizing enterocolitis remains imperfectly understood, it is considered multifactorial, with several contributing causes extensively analyzed over the past 40 years. Some factors are more frequently encountered in babies with necrotizing enterocolitis, thus constituting the classical triggering factors for necrotizing enterocolitis-like bowel damage ^16^. Prematurity is the most recognized predisposing factor for necrotizing enterocolitis and it rarely occurs in full-term infants ^17^, where it is usually secondary to congenital diseases, such as cardiac anomalies ^18, 19^. Conversely, the majority of infants with necrotizing enterocolitis are born preterm, and the risk of developing necrotizing enterocolitis is inversely related to gestational age and birth weight ^20– 21^. Prematurity implies immaturity of gut motility and digestion, intestinal circulatory regulation, gut barrier function, and immune defense. Another well-recognized predisposing factor is formula feeding. The mechanism of injury may be fluid shift from villus vessels to the bowel lumen, resulting in an ischemic insult to the mucosa. Therefore, not only hyperosmolar formulas but any hyperosmolar fluid, including oral medications using hyperosmolar vehicles or contrast media, could lead to mucosal injury in the bowel ^22, 23^. Hypoxia is a well-studied phenomenon that can lead to necrotizing enterocolitis: recurrent episodes of apnea, respiratory distress, assisted ventilation, and umbilical vessel catheterization, and can all contribute to hypoxic events in very low birth weight neonates ^24^. Disruption of commensal bacteria, leading to deficient or abnormal microbial colonization of the gut, has been implicated as a key risk factor in the pathogenesis of necrotizing enterocolitis ^25^. Whether bacterial infection has a primary inciting role in necrotizing enterocolitis or whether an initial intestinal mucosal injury allows secondary bacterial invasion is unclear. The most commonly identified organisms are: *Escherichia Coli*, *Klebsiella pneumoniae*, Proteus, *Staphylococcus aureus*, S. Epidermidis, Enterococcus spp., *Clostridium perfringens*, and *Pseudomonas aeruginosa.*

## Imaging

Plain radiography is the cornerstone of necrotizing enterocolitis diagnosis and staging ( Table 1) ^5, 6, 26^. The pathognomonic radiological finding of necrotizing enterocolitis is that of *Pneumatosis intestinalis*, defined as gas in the bowel wall originating from pathogenic bacteria. Other findings that are seen in more severe forms of necrotizing enterocolitis are ascites and portal venous gas. Pneumoperitoneum resulting from intestinal perforation is indicative of intestinal perforation and/or necrosis.

Other imaging modalities could provide more information and help diagnosis and follow-up of infants with necrotizing enterocolitis. Doppler ultrasonography, especially aimed at measuring blood flow velocity in the coeliac trunk and superior mesenteric artery, has been used to identify patients at risk of developing necrotizing enterocolitis, as well as to assess the bowel viability in those infants with established necrotizing enterocolitis ^27– 30^. In particular, sonographic findings of free gas, focal fluid collection, increased bowel wall echogenicity, absent bowel perfusion, portal venous gas, bowel wall thinning or thickening, and *Pneumatosis intestinalis*, are associated with an adverse outcome. Conversely, the use of magnetic resonance imaging scans in human necrotizing enterocolitis was described only once in the literature and remains anecdotal so far ^31^.

Near-infrared spectroscopy is a noninvasive real-time method of measuring local tissue oxygenation that is being evaluated as a predictive diagnostic modality for necrotizing enterocolitis. Gay *et al.* demonstrated that, in a piglet model of necrotizing enterocolitis, splanchnic tissue oxygenation measurements are directly correlated with changes in intestinal blood flow and markedly reduced by necrotizing enterocolitis ^32^. Patel *et al.* reported that abdominal near-infrared spectroscopy measurements are lower and have increased variability in preterm infants with necrotizing enterocolitis ^33^.

## Diagnosis and disease progression

Infants with necrotizing enterocolitis present with clinical symptoms of abdominal distension, feeding intolerance, bilious vomiting, and bloody stools, and with laboratory derangements characterized by neutropenia, thrombocytopenia, metabolic acidosis, and high C-reactive protein levels ^26^.

In some infants, necrotizing enterocolitis progresses to peritonitis, intestinal perforation, septic shock, disseminated intravascular coagulation, and death. However, the majority of patients that require surgical intervention do not present with a fulminant course, resulting in disease progression ^34^. Clinical parameters alone cannot accurately predict necrotizing enterocolitis progressing to surgical disease in over 40% of patients, and biomarkers have been used to evaluate disease extent and progression ^35^. Novel biomarkers of necrotizing enterocolitis have been described in serum, urine, feces, buccal swab, or using noninvasive hemodynamic technique (heart rate activity) ^35– 38^.

Several authors have reported the use of laparoscopy to diagnose necrotizing enterocolitis ^39– 42^. Pierro *et al.* reported that laparoscopy can provide important information regarding bowel viability, is feasible and tolerated even in infants weighing less than 1 kg, and can be safely performed on the intensive care unit ^39^. Moreover, Numanoglu and Millar reported that when bowel ischemia is suspected, fluorescein laparoscopy could be useful to identify the necrotic segments ^41^.

In 1956, Porter first described a 2-day-old infant who developed a spontaneous intestinal perforation (SIP) ^43^. SIP most commonly presents as pneumoperitoneum without pneumatosis and, according to some studies, it has distinct, non-ischemic histopathology, different from that of necrotizing enterocolitis ^44, 45^. Gordon *et al.* were the first to demonstrate the deleterious relationship between early postnatal steroids and SIP in a retrospective cohort ^46^. However, despite extensive literature on the subject, there has been an ongoing debate on whether this condition represents a mild form of necrotizing enterocolitis or a distinct entity. In the latter case, many studies on necrotizing enterocolitis could be “contaminated” by infants who instead had SIP.

## Medical management

Most infants with suspected (Bell’s stage I) or confirmed (Bell’s stage II) necrotizing enterocolitis are managed non-operatively. Non-operative treatment includes withholding feeds, ventilatory support, fluid resuscitation, inotropic support, correction of acid-base imbalance, coagulopathy and/or thrombocytopenia, bowel rest, and antibiotics.

Currently, there is no consensus and no evidence in the literature on which antibiotic regimen should be prescribed for medically managed infants with necrotizing enterocolitis, and this is reflected by both an international survey and a Cochrane review ^48, 49^. Therefore, antibiotics are prescribed depending on institutional protocol and changed according to individual culture and sensitivity results. Similarly, the duration of bowel rest with no enteral feeds for medically treated infants with necrotizing enterocolitis is based on tradition and not on evidence-based treatment ^48^.

## Surgical management

A proportion of medically managed infants with necrotizing enterocolitis require acute surgical intervention, due to clinical deterioration or intestinal perforation. Whilst the latter indication is clearly identified with radiologic evidence of pneumoperitoneum, signs of clinical deterioration leading to surgery can be more subtle. These include requirement of inotropes, worsening abdominal findings, hemodynamic instability, worsening laboratory values (intractable acidosis, persistent thrombocytopenia, rising leukocytosis, or worsening leukopenia), and/or sonographic evidence of decreased or absent bowel perfusion.

A laparotomy in high-risk neonates, especially if born with an extremely low weight, can result in serious morbidity or even mortality. To avoid this risk, in 1977 Ein *et al.* first described the percutaneous insertion of a peritoneal drain in five neonates with bowel perforation as a temporizing measure to delay laparotomy ^50^. The authors noticed a clinical improvement of these infants within a week, so that they advocated the peritoneal drainage of small infants with perforated necrotizing enterocolitis. In support of this approach, a few years later, the same authors published a bigger series where they showed that 40% of neonates <1500 g treated with the peritoneal drain had complete resolution of their disease without requiring further surgery ^51^. A similar experience with the peritoneal drain was later reported by other authors ^52– 54^. However, this surgical approach has been very controversial and two prospective randomized controlled trials comparing the use of peritoneal drain *vs.* laparotomy in infants with perforated necrotizing enterocolitis were carried out ^55, 56^. Interestingly, neither of the two trials was able to demonstrate an advantage of one treatment modality over the other ^55, 56^. Moreover, Rees *et al.* demonstrated that in neonates with <1000 g body weight and perforated necrotizing enterocolitis, peritoneal drainage was not a definitively effective procedure, as 74% of the infants required a rescue laparotomy ^57^. It is still debatable whether there is a role for peritoneal drainage in the stabilization of a critically unwell child with perforated necrotizing enterocolitis and/or respiratory compromise, prior to the transfer to another center for laparotomy ^57^.

The universal principles of surgery in necrotizing enterocolitis are to remove the necrotic intestine and control intra-abdominal sepsis while preserving as much intestinal length as possible ^57^. Within these principles, there are different surgical options that surgeons favor on the basis of personal experience, rather than evidence-based literature ^58^. The classical approach to necrotizing enterocolitis has been to resect all areas of the necrotic intestine and fashion a stoma to allow adequate time for healing and growth before restoring intestinal continuity at a later stage. However, stomas, and in particular jejunostomies, are poorly tolerated by preterm infants, as they predispose them to nutritional and metabolic disturbances and poor growth as a consequence of fluid and electrolyte depletion ^47^. Therefore, some surgeons would resect necrotic bowel and perform a primary anastomosis, even in neonates weighing less than 1000 g ^58^. To investigate which is the most effective operation for neonates with surgical necrotizing enterocolitis, a multicenter randomized controlled trial of resection with primary anastomosis *vs.* resection with stoma (STAT: Stoma or Intestinal Anastomosis Trial) is currently underway.

Moreover, there is no consensus among surgeons on the type of stoma to fashion and where to locate it with regard to the surgical wound ^58^. This is in line with the outcomes of a recent systematic review of the literature that showed no difference in the type or location of colostomy in children with colorectal disease ^59^.

At laparotomy, some infants are found to have multifocal necrotizing enterocolitis and require multiple resections and multiple anastomoses. In 1996, Vaughan *et al.* described an alternative approach for such cases: the “clip-and-drop” technique ^60^. According to this technique, the multiple necrotic areas are resected, the bowel ends are sealed with titanium clips or staples, and the clipped bowel loops are returned to the abdominal cavity. At a second-look laparotomy, the bowel loops can be reassessed and anastomoses can be performed. Since the first description, the “clip-and-drop” technique has been employed for infants with multifocal necrotizing enterocolitis by other authors ^60– 62^.

When the vast majority of the intestine is affected by severe intestinal damage, the patient is considered to have pancolitis or *NEC totalis*. This is a very controversial scenario, as the resection of the necrotic bowel may involve almost the whole intestine. Options include closing the abdomen and withdrawing care, or creating a diverting jejunostomy. The latter has been described to rescue a proportion of neonates with extensive necrotizing enterocolitis and to result in enteral autonomy in most patients ^63^.

## Outcome

Despite advancement in medical and surgical treatment over the last 6 decades, the mortality for necrotizing enterocolitis is still very high, especially in extremely low birth weight (ELBW) infants ^64^. Although the risk and absolute mortality of necrotizing enterocolitis decrease with higher birth weight, necrotizing enterocolitis has a relatively greater impact upon mortality at higher birth weight ^64^. Moreover, mortality for necrotizing enterocolitis is high even in cases of minimal bowel involvement ^65^.

Necrotizing enterocolitis survivors are also at high risk of developing severe complications, related either to the intestinal or to the systemic insult. Morbidities include recurrent episodes of necrotizing enterocolitis, development of intestinal strictures, intestinal failure, parenteral nutrition-related complications, and neurodevelopmental disabilities. About 10% of infants who have previously undergone surgery for necrotizing enterocolitis develop a recurrent episode and this results in long-term parenteral nutrition dependency ^66^. About a quarter of patients who have had necrotizing enterocolitis, especially if treated surgically, will develop one or more intestinal strictures ^67, 68^. Such strictures are investigated with contrast studies, typically in the form of enemas, due to the higher incidence in the colon, and usually require surgical resection. Strictures are the result of a vascular occlusion or spasm following the initial ischemic episode, as confirmed at histology by Kosloske *et al.* ^68^. In this seminal paper, Kosloske *et al.* reported the death of an infant due to the late diagnosis of post-necrotizing enterocolitis strictures and recommended a barium enema for all infants who had necrotizing enterocolitis about 6 weeks after the acute episode ^68^.

Longer-term outcome is related to the remaining intestinal length and its capacity for adequate nutrient absorption. The incidence of intestinal failure among infants undergoing surgical treatment for necrotizing enterocolitis is high, and many factors characteristic of severe necrotizing enterocolitis (such as low birth weight, antibiotic use, ventilator use, and greater extent of bowel resection) are associated with the development of intestinal failure ^69^. However, according to a recent study on a large cohort of children with intestinal failure one year after diagnosis, the diagnosis of necrotizing enterocolitis proved to be a significant predictive factor of enteral autonomy, possibly due to the greater capacity for late adaptation than the residual intestine after resection for other etiologies ^70^.

Finally, it is being increasingly recognized that approximately 50% of the neonates who developed necrotizing enterocolitis have a deleterious neurodevelopmental effect, although the mechanisms by which this develops is still poorly understood ^14, 71^. It is known that infants with necrotizing enterocolitis are at greater risk of motor impairment, and this seems to be mediated by white matter abnormalities on magnetic resonance imaging at term ^72^.

## Emerging strategies

Efforts to improve necrotizing enterocolitis outcome are directed towards prevention and treatment of the disease.

### Prevention

Probiotics are live microorganisms that increase natural intestinal defenses by regulating inflammatory response, cellular proliferation, and apoptosis. Several studies have demonstrated the efficacy and safety of prophylactic enteral probiotic administration in the prevention of necrotizing enterocolitis in infants with very low birth weight. A Cochrane review on this topic analyzed 24 trials and demonstrated that enteral probiotics supplementation significantly reduced the incidence of severe necrotizing enterocolitis and mortality ^73^. The probiotics preparations that were found to be effective contained lactobacillus either alone or in combination with bifidobacterium ^73^. New studies on probiotics in necrotizing enterocolitis are now directed to assess the most effective preparations, timing, and length of therapy to be utilized.

Other preventive strategies are to supplement formulas with prebiotics or synbiotics. Prebiotics are indigestible fiber compounds that stimulate the activity and growth of healthy bacteria within the intestine, whereas synbiotics are a combination of both prebiotics and probiotics, which exert a synergistic effect. Several studies report the beneficial effect of prebiotics or synbiotics on necrotizing enterocolitis incidence in preterm infants with variable outcomes ^74– 77^.

### Treatment

Novel treatment strategies have been tested in experimental models of necrotizing enterocolitis. These include Captopril ^78^, platelet-activating factor antagonists ^79^, heparin-binding epidermal growth factor-like growth factor ^80^, granulocyte colony-stimulating factor and erythropoietin ^81^. One promising maneuver that was initially tested in animals and then confirmed in a safety and feasibility trial on human infants is moderately controlled hypothermia ^82– 87^. In animal studies, controlled hypothermia resulted in prolonged survival, prevention of liver energy failure, reduction in neutrophil infiltration of lungs and intestine, attenuation of the derangement in cardiac oxidative metabolism, attenuation of histological damage to the intestine and attenuation of the pro- and anti-inflammatory cytokine response in the portal vein and the systemic circulation ^82– 86^. In the first trial on preterm neonates with severe necrotizing enterocolitis, controlled hypothermia proved to be feasible and safe for 48 hours ^87^.

Stem cell therapy, which is nowadays a therapeutic option for refractory inflammatory bowel disease ^88^, has recently been proposed as a novel strategy for infants with necrotizing enterocolitis. In a neonatal rat model of necrotizing enterocolitis, amniotic fluid stem cells, injected intraperitoneally, proved to integrate into the bowel wall, improve survival, reduce necrotizing enterocolitis incidence, decrease gut damage and improve intestinal function ^89– 92^. Amniotic fluid stem cell administration was associated with the migration of cyclooxygenase-2 (COX-2) expressing stromal cells from the lamina propria of the small intestinal villi to a position near the base of the intestinal crypts ^90^. The beneficial effects of amniotic fluid stem cells on the development of necrotizing enterocolitis were blocked by the administration of a selective COX-2 inhibitor, suggesting that the migration of COX-2 cells was involved in the protective effects of the amniotic fluid stem cells. Moreover, the same beneficial effect exerted by amniotic fluid stem cells on rat survival was also obtained when a conditioned medium (supernatant) from amniotic fluid stem cells was administered ^90^. These findings suggest that amniotic fluid stem cells act *via* a paracrine mechanism, by secreting factors that stimulate bowel regeneration. Future studies, aimed at identifying those factors secreted by amniotic fluid stem cells, could pave the way for a novel pharmacological therapy for infants with necrotizing enterocolitis.
